# Experimentally guided computational antibody affinity maturation with *de novo* docking, modelling and rational design

**DOI:** 10.1371/journal.pcbi.1006980

**Published:** 2019-05-01

**Authors:** Daniel A. Cannon, Lu Shan, Qun Du, Lena Shirinian, Keith W. Rickert, Kim L. Rosenthal, Martin Korade, Lilian E. van Vlerken-Ysla, Andrew Buchanan, Tristan J. Vaughan, Melissa M. Damschroder, Bojana Popovic

**Affiliations:** 1 Department of Antibody Discovery and Protein Engineering, AstraZeneca, Cambridge, United Kingdom; 2 Department of Antibody Discovery and Protein Engineering, AstraZeneca, Gaithersburg, Maryland, United States of America; 3 Department of Oncology Research, AstraZeneca, Gaithersburg, Maryland, United States of America; Fox Chase Cancer Center, UNITED STATES

## Abstract

Antibodies are an important class of therapeutics that have significant clinical impact for the treatment of severe diseases. Computational tools to support antibody drug discovery have been developing at an increasing rate over the last decade and typically rely upon a predetermined co-crystal structure of the antibody bound to the antigen for structural predictions. Here, we show an example of successful *in silico* affinity maturation of a hybridoma derived antibody, AB1, using just a homology model of the antibody fragment variable region and a protein-protein docking model of the AB1 antibody bound to the antigen, murine CCL20 (muCCL20). *In silico* affinity maturation, together with alanine scanning, has allowed us to fine-tune the protein-protein docking model to subsequently enable the identification of two single-point mutations that increase the affinity of AB1 for muCCL20. To our knowledge, this is one of the first examples of the use of homology modelling and protein docking for affinity maturation and represents an approach that can be widely deployed.

## Author summary

The role of computational techniques in therapeutic protein development is multifaceted and includes structure prediction (homology modelling), interface identification (docking), and mutational energy change calculation. Success has been reported in the areas of protein structure prediction and interface prediction (see competition results such as Critical Assessment of Structure Prediction [CASP] and Critical Assessment of Predicted Interactions [CAPRI]), but perhaps one of the greatest challenges is the translation of *in silico* derived binding energy changes upon mutation into affinity matured antibody variants. In these applications, it is important to choose the correct structural models, or approximations, that make sense across all aspects of *in silico* protein design. The challenges are compounded when no antibody-antigen co-crystal structure is available and there is a high degree of uncertainty around the protein-protein interface. Although the field is arguably far from its goal of precisely correlating computational predictions with experimental data, we show that even in the absence of a co-crystal structure, it is possible to identify modest affinity-improving mutations by using *in silico* mutagenesis in combination with homology modelling, protein docking, and simple experimental checkpoints.

## Introduction

Antibodies are the most specific class of binding molecules known and their versatility has led to many successful therapeutics for the treatment of severe diseases. Structurally, antibodies are multi-domain proteins formed by beta-sheets that are held together by disulfide bridges. Two immunoglobulin domains, the variable light chain (V_L_) and the variable heavy chain (V_H_) domains, are joined together to create the variable fragment (Fv). Wu and Kabat’s original works [1] identified six hypervariable regions on the V_H_ and V_L_ domains and correctly predicted that such regions are responsible for the specific binding of the antigen. These loops, the complementarity-determining regions (CDRs), arise from a relatively conserved framework region (FR) and are typically in close spatial proximity to the antigen. The V_L_ and V_H_ domains together generate a binding site for the antigen that is in large part mediated by CDRs.

Antibody discovery platforms use either a display-based library approach (phage, yeast, ribosome, mammalian, or other systems) or an immunisation and hybridoma screening strategy for antibody isolation. Once a panel of lead antibodies has been isolated, their binding affinity often requires optimisation if the antibody is to be a potential therapeutic. The display methods mentioned above can be used for *in vitro* affinity maturation because they allow for control of antigen concentration, presentation format, and deselections to eliminate unwanted specificities. These methods, along with other random mutagenesis methods, have proven very successful for affinity improvements [2–7]. However, the process of *in vitro* affinity maturation can be laborious and time consuming, taking many months, and more efficient methods to improve affinity would be beneficial.

A number of strategies for *in silico* antibody affinity maturation have been reported, typically employing either a structure-based rationale [8–11] or a mini-library approach [12]. The success of these methods hinges primarily on two factors: first, the presence of a high-quality co-crystal structure, and second, an algorithm to calculate the energy change that occurs upon mutation.

Application of free energy perturbation (FEP) [13–15] and potential-of-mean force (PMF) [16] methods to predict free energy changes in proteins has been reported in the literature [17–20]. However, they often require significant computational time and cost, which significantly limit their application for *in silico* antibody lead optimisation. More commonly, methods based on molecular mechanics (MM), coupled with an implicit solvent model such as generalized Born surface area (MM-GBSA), molecular mechanics-Poisson-Boltzmann surface area (MM-PBSA) [21, 22] or the Lazaridis-Karplus solvation model (MM-LKSM), are employed to estimate free energies with significant savings in computing time and infrastructure [23–26], allowing thousands rather than dozens of mutations to be calculated. The disadvantage of using molecular mechanics calculations is that they do not account for global conformational changes that may arise upon mutation, resulting in ΔΔE values that are not truly representative of the real protein. This is reflected in the poor correlation between MM-GBSA/MM-PBSA/MM-LKSM free energy changes and experimental data, which is often less than 0.5 (Pearson correlation) [27, 28].

The possession of high-quality antibody structural information is widely considered to be an essential starting point for computational antibody affinity maturation. To our knowledge, there have been few published reports of *in silico* affinity maturation in which the co-crystal structure of the antibody-antigen complex is not available. In this scenario, affinity maturation is particularly challenging due to the many assumptions and errors that are propagated during computational calculations and simulations of large biological systems, especially if the wild-type antibody is already a strong binder with K_D_ in the nanomolar range. Despite these challenges, we have decided to test such an approach, choosing to build a homology model of an Fv fragment and to use the nuclear magnetic resonance (NMR) structure of the antigen for protein-protein docking. We optimised the protein-protein docking with experimental guidance from a panel of alanine scanning mutations in the VH and VL domains and used the *in silico* complex to drive *in silico* affinity maturation. In this paper, we are the first to describe the application of such an approach to obtain affinity and activity gains for a high affinity antibody that, although relatively modest, demonstrate the potential of this methodology.

## Results

### Homology modelling and protein-protein docking of murine CCL20-AB1

#### Structure preparation

**Fig 1:**
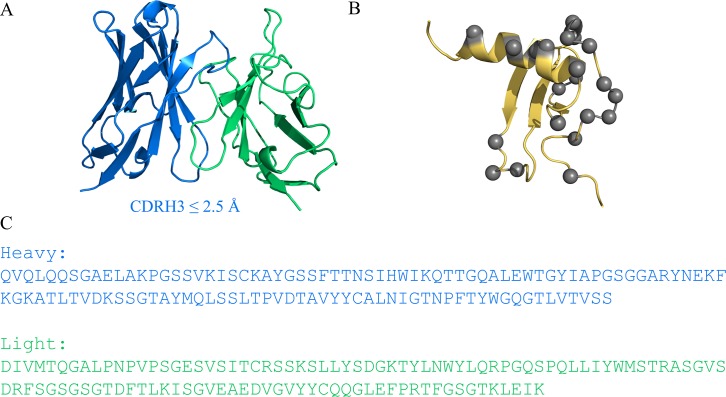
(A) Homology model of AB1 antibody with V_H_ (blue) and V_L_ (green) domains, annotated with the model-predicted accuracy on the CDR H3 at ≤2.5 Å. (B) Structure of muCCL20 showing human and murine sequence differences (grey). (C) V_H_ (blue) and V_L_ (green) sequences of anti-muCCL20 antibody AB1.

The murine protein CCL20 (muCCL20) and its binder, AB1, were chosen for our test case because they fit the criteria of nanomolar-range binding affinity as well as reagent and assay availability. Since the only available crystal structure was that of the antigen but not of the antigen-antibody complex, we had the opportunity to investigate the potential of *de novo* affinity improvement from homology modelling. The AB1 Fv model was generated with SabPred [29] (Fig 1A), and the structure of muCCL20 was sourced from the Protein Data Bank (PDB ID, 1HA6) and used for docking (Fig 1B).

Protein-protein docking was used to establish a putative complex between CCL20 and the AB1 Fv region. The docking procedure generated 54,000 poses in total, the top 2,000 of which were carried forward for refinement. These 2,000 poses were refined with the RDOCK algorithm [29]. The five top ranked poses by RDOCK score were contained within three structural clusters; each of the top five poses had an RDOCK score of less than –30.00, and the best pose had a score of –36.20 (Table 1). Solvent accessible surface area (SASA) was used as a measure of surface area buried upon binding.

**Table 1:** Details of the five highest-ranked, by RDOCK score, rigid-body docking poses.

Rank	Pose	Cluster	RDOCK score	Contact SASA (Å^2^)
1	1904	1	–36.20	966.44
2	1704	1	–35.31	957.56
3	1843	1	–34.09	1,002.44
4	1334	6	–33.39	897.13
5	1644	2	–31.52	705.93

*SASA stands for solvent accessible surface area.

**Fig 2:**
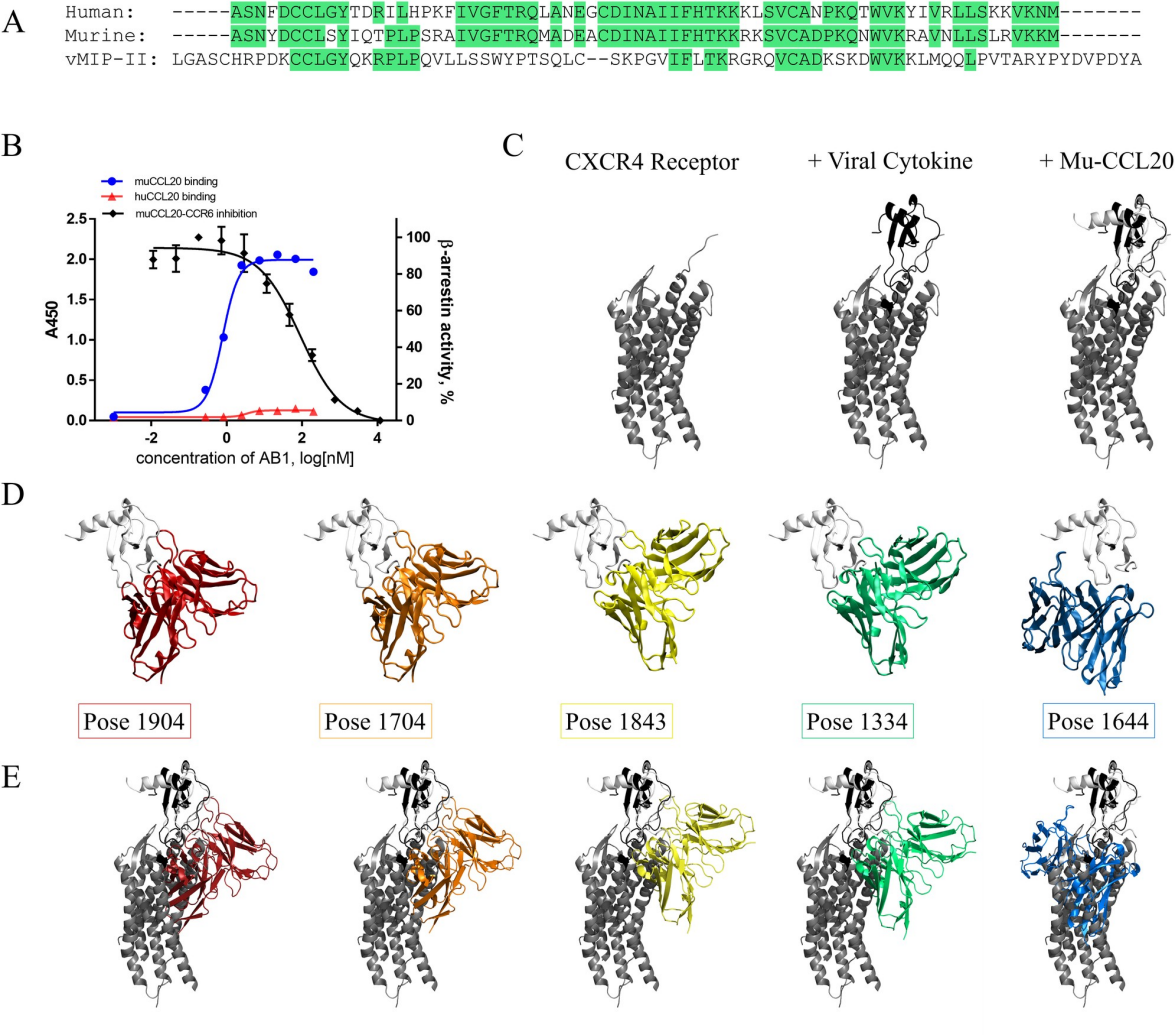
(A) Sequence alignment of human and mouse CCL20 with vMIP-II; identical residues are highlighted in green. (B) Binding specificity of AB1 antibody to murine (blue line) but not human (red line) CCL20. AB1 also blocked the interaction between muCCL20 and its receptor muCCR6 (black line). (C) Structural alignment of muCCL20 (white) with the CXCR4 (grey) and vMIP-II (black) co-crystal structure, 4RWS. (D) Top five highest-ranked antibody-muCCL20 docked poses, 1904 (red), 1704 (orange), 1843 (yellow), 1334 (green), and 1644 (blue), (E) aligned to vMIP-II in the surrogate receptor ligand complex.

The antigen in our case study, muCCL20, shares 67% sequence homology with the human orthologue (Fig 2A). In comparison, the sequence identity between muCCL20 and viral macrophage inflammatory protein 2 (vMIP-II) is 30.65% and the sequence similarity is 48.39%. We performed binding enzyme-linked immunosorbent assays (ELISAs) and found that our antibody, AB1, did not bind human CCL20 (Fig 2B), which provided us with key data to inform the determination of feasible docking poses. In addition, we performed a cell-based activity assay in which the activity conferred through the interaction of muCCL20 with its cell surface–expressed receptor CCR6 was blocked by AB1. Hence, it was possible to further narrow the docking search space to include only those poses that blocked this interaction.

At the time of this writing, no crystal structure of either human or murine CCR6 was available, either as the receptor alone or in complex, making the assessment of stereospecific blocking of ligand-receptor interactions challenging. To guide docking experiments, we therefore inferred structural knowledge from a surrogate complex. Both the receptor and chemokine folds are strongly conserved within the CXCR receptor family, so we used the co-crystal complex of another receptor-ligand family member, human CXCR4 and vMIP-II (PDB ID, 4RWS), as a template for the conformation of the CCR6-CCL20 complex (Fig 2C). The root mean squared deviation of the two structures was found to be 2.35 Å with the jFATCAT [30] (rigid) alignment algorithm.

Each of the top docked poses were aligned with the CXCR4–vMIP-II complex to determine whether the relative antibody-antigen conformations (Fig 2D) satisfied the criteria of stereospecifically blocking cytokine-receptor binding (Fig 2E). In each of the five docked poses, the AB1 Fv was overlaid with the receptor, indicating that these poses would potentially block the interaction of CCL20 with the CCR6 receptor.

**Fig 3:**
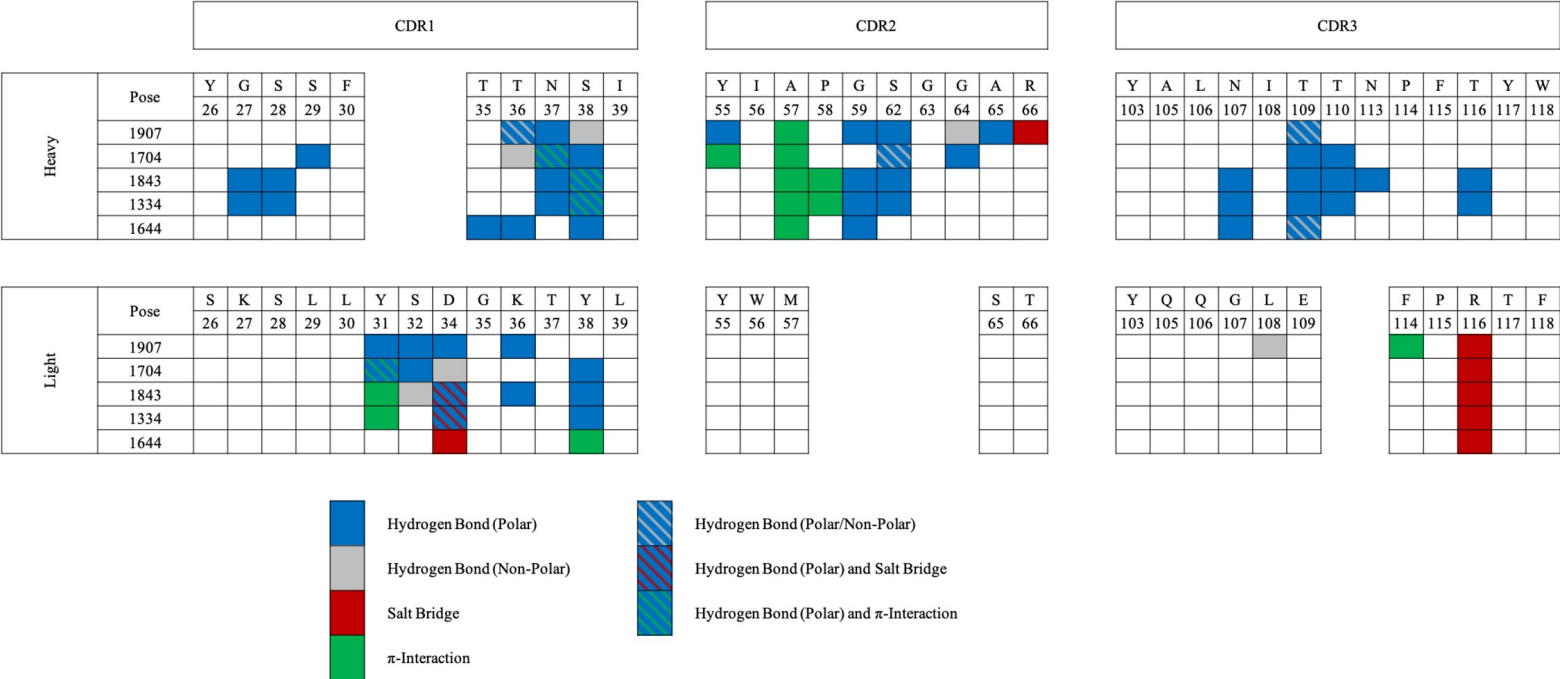
Predicted intermolecular interactions between antibody and antigen for each of the five candidate poses.

To understand the binding properties of each pose, we performed interfacial analysis and determined the numbers of explicit intermolecular interactions between the antibody and antigen (Fig 3). Both the top-ranked pose, 1904, and the third-ranked pose, 1843, displayed the greatest number of intermolecular contacts. Mutations to human CCL20 from muCCL20 were performed simultaneously and resulted in loss of binding affinity for each pose (S1 Table); the greatest relative loss was attributed to pose 1843, which had the greatest number of total intermolecular interactions (+2.57 arbitrary units [AU]). The anti-muCCL20 antibody is not cross-reactive with the human antigen, so to further assess the validity of the top five poses, we performed *in silico* mutagenesis of the muCCL20 in which the resulting “species switch” would be predicted to result in a loss of affinity of the antibody.

#### Refining the structural model of AB1 by alanine scanning *in silico*

**Fig 4:**
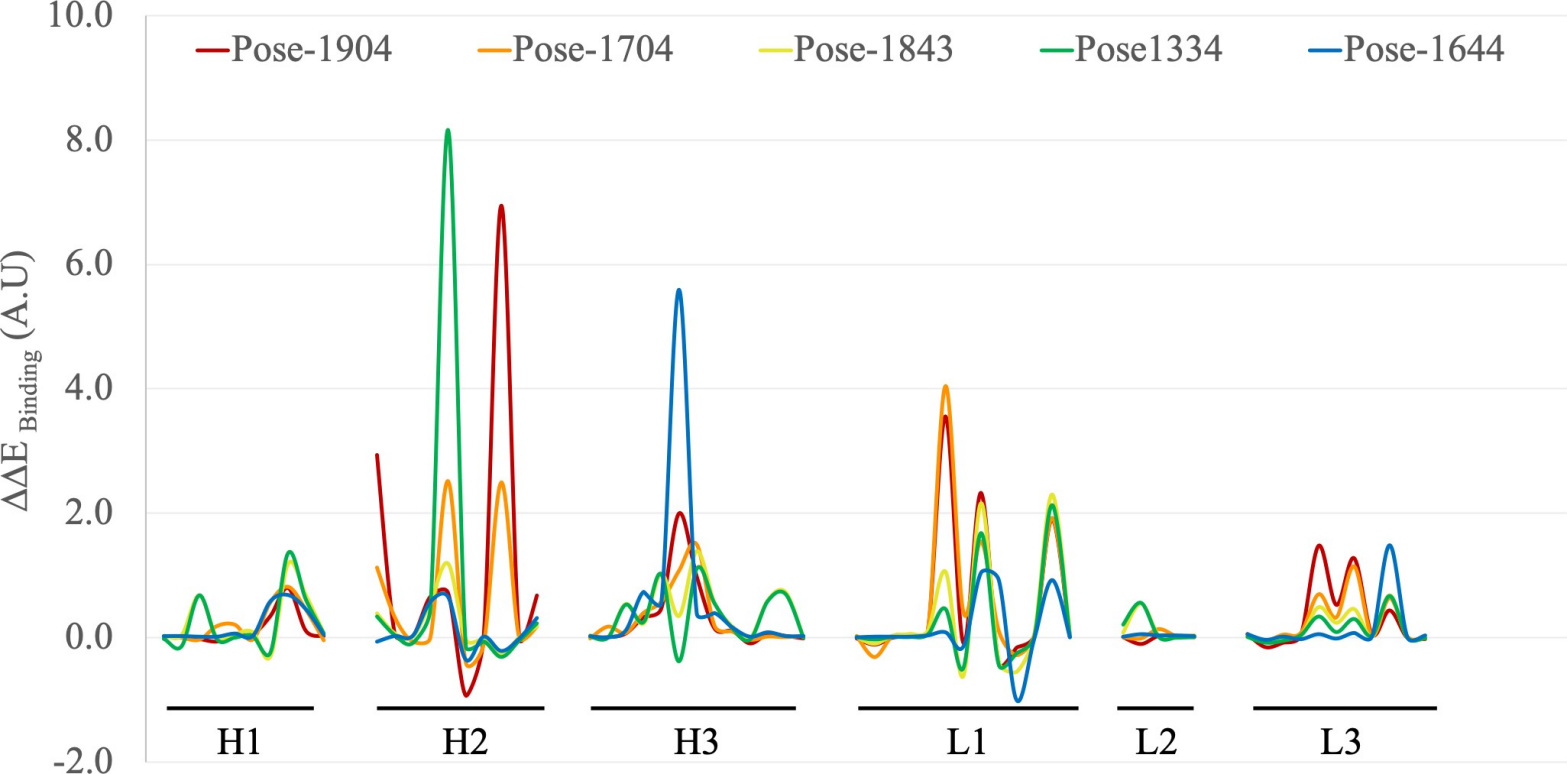
*In silico* alanine scanning mutagenesis was completed for all five poses. Poses 1904, 1704, 1843, 1334 and 1644 are shown in red, orange, yellow, green and blue respectively. The graph shows relative ΔΔE_binding_ changes upon alanine mutation for the five candidate docked poses across the IMGT-defined CDR regions.

We performed *in silico* alanine scanning of the top five poses to further refine the models by identifying residues that are important for affinity in the context of differing protein-protein binding conformations. Each of the five poses gave a unique alanine scanning profile (Fig 4), a feature that was exploited to experimentally validate the docking procedure and to identify the docked pose that most closely resembled the true complex conformation. The panel of mutants chosen for experimental validation was based on three criteria: they had to (1) be contained within the CDR (international ImMunoGeneTics information system [IMGT] [31]) loops, including Vernier residues; (2) be involved in a unique, explicit intermolecular interaction between the antibody and antigen; or (3) present a significant difference in ΔΔE_binding_ value upon mutation to alanine when compared with at least two of the other candidate poses.

Using these criteria, we identified four single-point mutations for which an explicit intermolecular interaction was predicted between the antibody and antigen and was unique to one of the five poses. Nine additional single-point mutations were selected, and five triple mutations were chosen as a combination of single mutations that presented a significant difference in ΔΔE_binding_ value for each pose. Finally, two negative control mutations, one on each antibody chain, in which there was no change in ΔΔE_binding_ value upon alanine mutation were selected, giving a total panel of 20 variants (Table 2).

**Table 2:** Relative ΔΔE_binding_ values for each proposed variant across the five docked conformations. IMGT numbering. Stabilizing mutations have negative ΔΔE_binding_ values; destabilizing mutations have positive ΔΔE_binding_ values.

	Variant	Mutations	Pose1904	Pose1704	Pose1843	Pose1334	Pose1644
**Unique**	1	H:S29A	–0.07	0.18	0.00	–0.03	0.01
	2	H:T35A	0.05	–0.04	0.09	0.03	0.02
	3	H:R66A	0.67	0.17	0.18	0.23	0.31
	4	H:N113A	0.14	0.18	0.56	0.55	0.39
**Triple**	5	H:Y55A, H:R66A, L:Y31A	4.02	4.19	1.29	0.34	0.34
	6	H:S29A, L:Y31A, L:Y38A	4.93	4.78	3.03	2.16	1.09
	7	H:S28A, H:N113A, L:K36A	–1.52	–1.02	0.24	–0.33	–1.62
	8	H:T110A, L:Y31A, L:D34A	6.72	6.93	5.20	3.70	2.62
	9	H:T35A, H:N107A, L:R116A	–0.98	–0.41	0.16	–0.29	0.31
**Singles**	10	H:S28A	-0.03	–0.04	0.68	0.68	0.01
	11	H:Y55A	2.93	1.12	0.39	0.34	–0.07
	12	H:N107A	0.32	0.39	0.23	0.25	0.73
	13	H:T110A	0.97	1.49	1.39	1.11	0.38
	14	L:Y31A	3.55	4.04	1.06	0.46	0.08
	15	L:D34A	2.32	1.54	2.16	1.68	1.04
	16	L:K36A	–0.17	–0.29	–0.55	–0.25	–1.01
	17	L:Y38A	1.90	1.92	2.30	2.13	0.92
	18	L:R116A	0.43	0.63	0.68	0.67	1.48
**Controls**	19	H:Y26A	0.01	0.01	0.03	–0.02	0.02
	20	L:S26A	–0.02	0.02	0.01	0.01	0.00

### Experimentally guided refinement of protein-protein docking of murine CCL20-AB1

#### Experimental identification of key antigen-antibody contact residues

**Fig 5:**
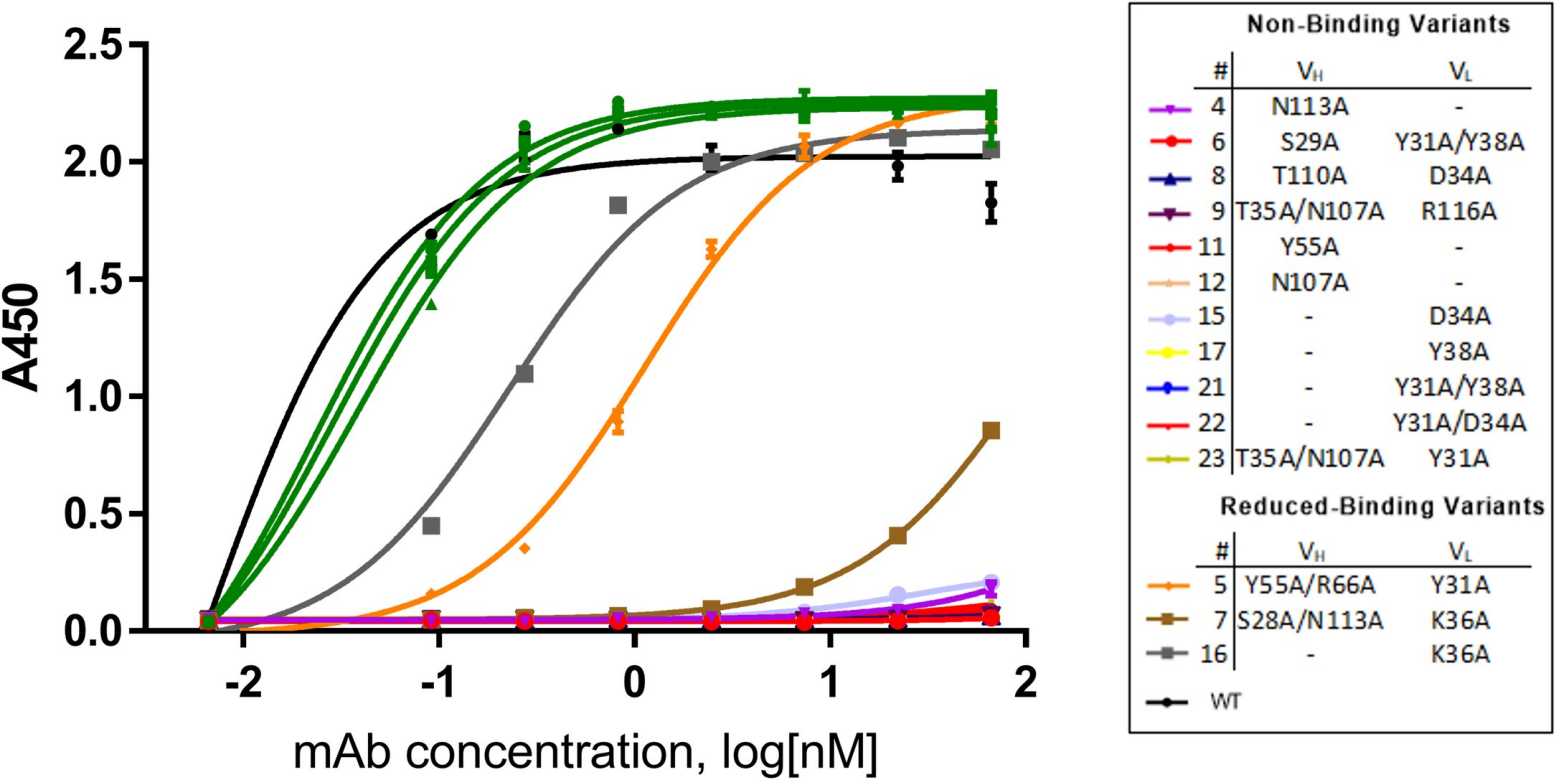
ELISA binding assay results representing the 23 alanine variants in comparison with WT AB1. The WT AB1 mAb is shown in black. Nine variants that retained binding are represented in green. As labelled in the inset, 11 variants (variants 4, 6, 8, 9, 11, 12, 15, 17, 21, 22, 23) were found to be non-binding and 3 variants had reduced binding (variants 5, 6, 17). The mutations in these variants are denoted in the inset.

Site-directed mutagenesis was carried out to generate the alanine mutations for possible key residues involved in antibody-antigen interaction. A total of 23 variants were generated, comprising the 20 variants detailed in Tables 2 and 3 additional variants generated as intermediate constructs during the combination of V_H_ and V_L_ mutations (variant 21, L:Y31A/Y38A; variant 22, L:Y31A/D34A; and variant 23, H:T35A/N107A/L:Y31A) (Fig 5). To distinguish the binding differences among these mutants, we assessed antigen binding with capture ELISA to better approximate 1:1 binding, that is, using plate-coated anti–fragment crystallizable (Fc) antibody to capture the panel of antibody variants for muCCL20 binding. Of the panel of 23 variants, 11 showed a complete loss in binding to the muCCL20 antigen and 3 (variant 5, 7, and 16) showed reduced binding, whereas the rest of the variants retained binding. Identification of the non-binding variants that were single-point mutations helped us to dissect the key residues involved in the binding interaction, and the double and triple mutations served as additional confirmation. Deconvolution of the results revealed that five positions, H:Y55, H:N107, H:N113, L:D34, and L:Y38, were essential for antigen binding, as alanine replacement resulted in complete loss of signal, and that the position L:K36 was also involved, as the alanine mutation reduced binding affinity by threefold (Fig 5).

**Table 3:** Panel of 20 single-mutation variants selected for affinity maturation of the anti-muCCL20 monoclonal antibody. Mutations 2, 10, 14 and 16 were selected from Discovery Studio; 1, 3, 4, 5, 7, 8, 11, 12, 13, 17, 18 and 19 from Schrödinger; 5, 6, 9 and 15 from Rosetta and the neutral control mutation is 20.

Variant	Chain	Parent	IMGT	Mutation	Variant	Chain	Parent	IMGT	Mutation
1	H	S	28	R	11	H	N	113	R
2	H	S	29	D	12	H	F	115	H
3	H	T	36	Q	13	H	T	116	R
4	H	N	37	T	14	L	S	32	D
5	H	G	59	Y	15	L	D	34	Q
6	H	G	64	T	16	L	G	35	Y
7	H	A	65	Y	17	L	K	36	R
8	H	I	108	R	18	L	Y	38	R
9	H	T	110	W	19	L	F	114	R
10	H	T	110	F	20	L	Q	106	Y

Comparison of binding ELISA data with *in silico* mutagenesis results found that none of the five candidate poses completely aligned with the experimental data. For each of the 14 variants that showed reduced or no binding, up to 18 intermolecular interactions were lost. Structural analysis was performed on each pose to determine whether a particular intermolecular interaction between the antibody and antigen was lost. Pose 1843 showed the greatest correlation; 16 of the 18 intermolecular interactions were lost upon alanine mutation, and interactions in poses 1334 (13 interactions), 1704 (11 interactions), 1904 (9 interactions), and 1644 (8 interactions) correlated less with experimental data. The implication of these findings is that, although pose 1843 may indeed be closest to the native structure, it does not satisfy all the “non-binding” variant criteria and is thus unreliable for use in *in silico* affinity maturation. Therefore, to improve the reliability of the structural model, we redocked the antibody and antigen, using these experimental mutagenesis data to guide the design.

#### Redocking of antibody and antigen

Protein docking was repeated, and the residues H:Y55, H:N107, H:N113, L:D34, L:K36, and L:Y38 were defined as interfacial within 5.0 Å of the antigen. The 1,925 poses generated were subsequently refined. Rather than simply selecting the highest-ranked poses by score, single alanine mutations were performed on each pose to identify those which matched the experimental binding profile of the mutations that had resulted in a reduction or loss of binding. A panel of 10 single alanine mutations—5 non-binding and 1 reduced binding variant (H:Y55A, H:N107A, H:N113A, L:D34A, L:K36A, and L:Y38A) and 4 controls (H:R66A, H:T110A, L:Y31A, and L:R116A)—were designed into all 1925 docked poses. From the *in silico* mutagenesis experiments, the ΔΔE_binding_ values were compiled and filtered so that poses were accepted only if the six deleterious mutations had ΔΔE_binding_ values of ≥0.00 AU and the four neutral mutations had ΔΔE_binding_ values of ≤0.50 AU. Only 8 of the 1925 conformations matched all criteria (S2 Table).

**Fig 6:**
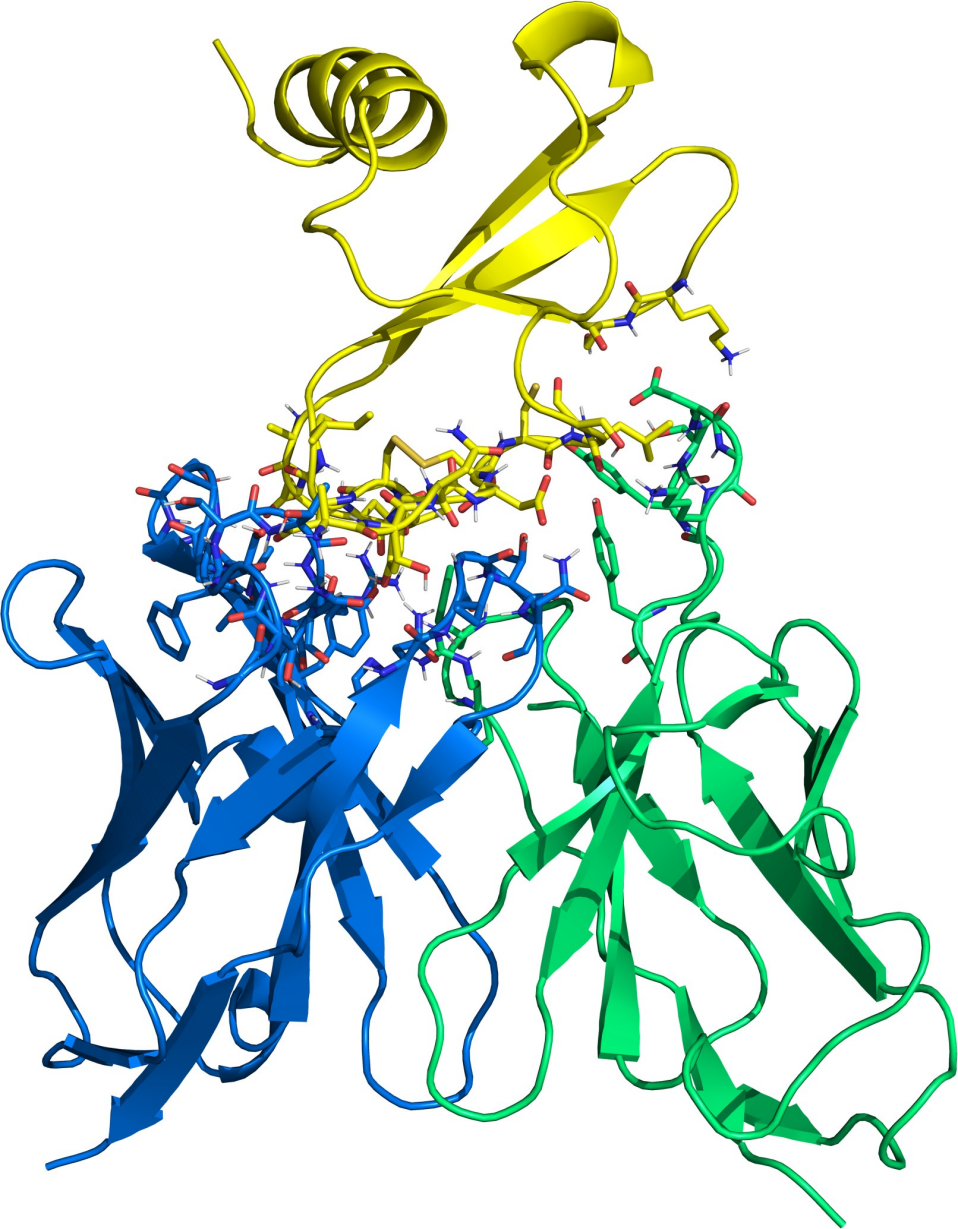
Re-docked protein complex of muCCL20 (crystal structure in yellow) and anti-muCCL20 antibody AB1 (homology model Fv, heavy chain in blue and light chain in green). Interfacial residues (5.0 Å) are shown in sticks representation.

Because the L:K36A mutation results in reduced rather than a complete loss of binding, the relative ΔΔE_binding_ value should be smaller than that of other deleterious mutations. Pose 491, which was ranked 120th overall, was found to match this profile more closely than any of the others we identified. This model was -subsequently chosen for *in silico* affinity maturation (Fig 6).

### Affinity maturation of AB1 through *in silico* design and experimental validation

#### *In silico* affinity maturation

To maximise the chance of identifying mutations that would improve antibody-antigen binding affinity, we used three separate *in silico* algorithms to perform the mutagenesis: Discovery Studio 2016 [32], Schrödinger Biologics Suite 2016–3 [33], and Rosetta [34]. The designable residues were limited to a hybrid selection of those of Kabat and colleagues [1] and IMGT [31] CDR residues to maximize sequence space in the variable region while avoiding framework residues. The CDRs were defined as CDRH1:G27-H40, CDRH2:Y55-G74, CDRH3:A105-Y117, CDRL1:R24-N40, CDRL2:W56-S69, and CDRL3:Q105-T117 (IMGT numbering).

To ensure that only the top mutations were identified, mutations in both Discovery Studio and Schrödinger Biologics Suite were filtered on the basis of a ΔΔE_binding_ value of ≤1.0 AU and a ΔΔE_stability_ value of ≤0.5 AU. Of the 1,400 Discovery Studio mutations, 46 met the criteria, having ΔΔE_binding_ values ranging from -2.46 to -1.02 AU. The filters were applied to both sets of Schrödinger mutations (0.0- and 5.0 Å optimisation radii), and 87 mutations for the 0.0 Å protocol and 154 mutations for the 5.0 Å protocol were accepted. A consensus of both protocols revealed 28 mutations that were common to both, having ΔΔE_binding_ values ranging from -18.75 to -1.12 for the 0.0 Å optimisation radius cutoff, and -12.13 to -1.20 for the 5.0 Å cutoff. From the site-by-site optimisation protocol of Rosetta, 29 sites were found to have acceptable mutations. The binding energy values were calculated by subtracting the wild-type ΔE_binding_ value from the mutant ΔE_binding_ value and identifying the most stabilising mutation. In addition, each accepted mutation was cross-checked with the point mutation stabilisation results to ensure that the mutation did not destabilise the complex, and ultimately 19 mutations were identified.

Of the 93 potential mutations that were identified across the three methods, 6 duplicates were identified, reducing the panel to 87. A panel of 20 mutations (Table 3) was selected for construction in the laboratory for empirical testing. These 20 included a diverse range of interfacial positions across both the V_H_ and V_L_ domains and included the most potentially beneficial mutations across each of the three methods employed. We also checked that no predicted thermodynamically stable water molecules were excluded. The final panel comprised 4 mutations identified with Discovery Studio, 11 with Schrödinger, 4 with Rosetta, and 1 control that was predicted to have no impact according to all three methods.

#### Binding and activity measurements to identify affinity-matured variants

**Fig 7:**
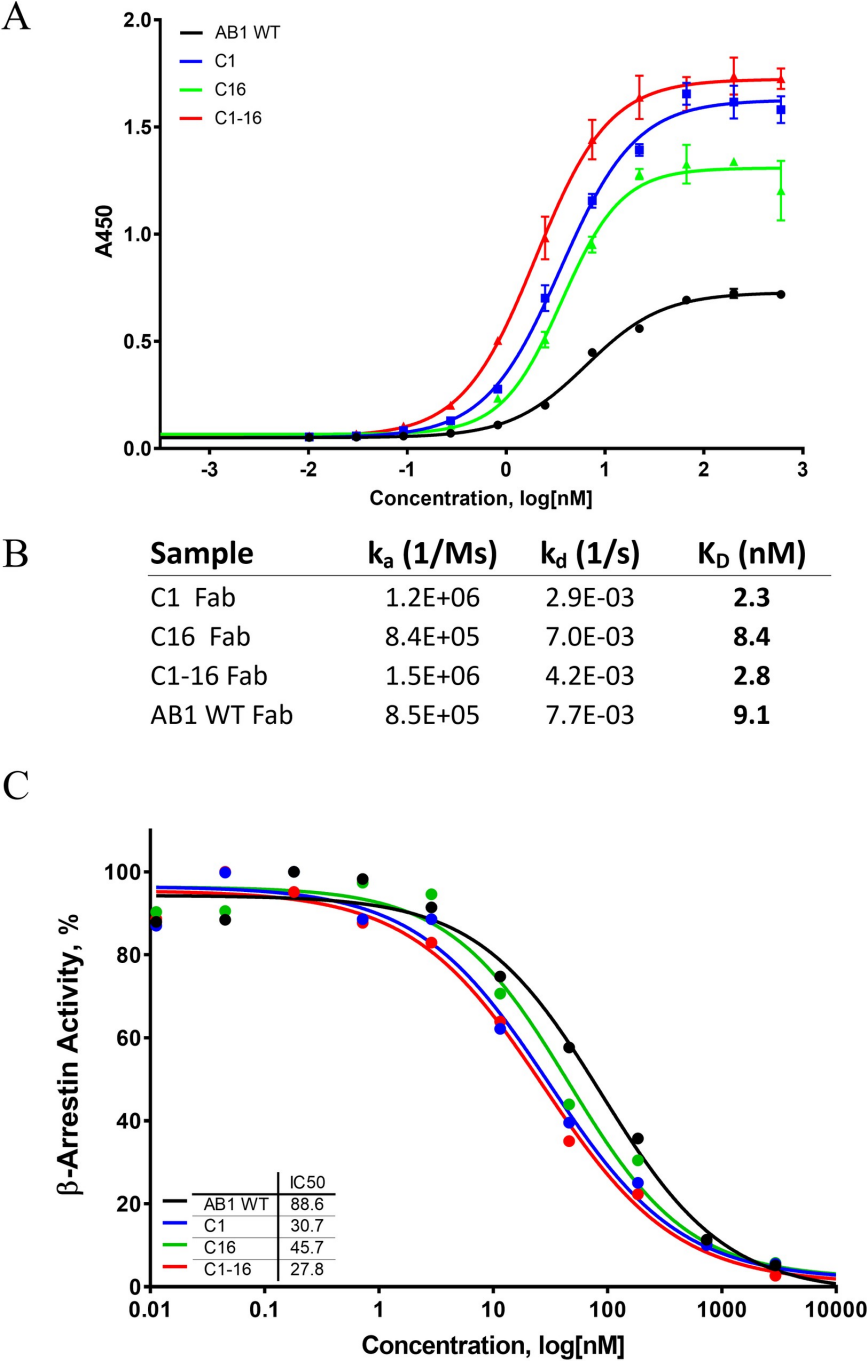
(A) ELISA revealed improved antigen binding with V_H_ clone 1 and V_L_ clone 16, as well as the combination clone with both variable domains. (B) Fab fragments of C1, C16, and C1-16 showed improved K_D_ values for muCCL20 when compared with parental AB1. (C) A cell-based β-arrestin assay demonstrated that the affinity-matured variants led to improved CCR6 receptor blocking activity when compared with the parental AB1 clone.

The panel of 20 predicted variants, 13 point mutations in the V_H_ domain and 7 point mutations in the V_L_ domain, were constructed by using site-directed mutagenesis and were subsequently expressed and purified. Capture ELISAs revealed that two of the variants, clone 1 (or AB1-C1, H:S28R) and clone 16 (or AB1-C16, L:G35Y), showed a noticeable shift in the binding EC_50_, whereas the rest of the mutants either retained or reduced binding when compared with the parent. We followed up by combining both the V_H_ and V_L_ mutations into a combination clone, clone 1–16 (or AB1-C1-16), and saw that it maintained the left shift in the binding curve (Fig 7A). To further confirm that these variants indeed conferred improved binding affinities, we proceeded to generate Fab fragments from these purified antibodies with papain proteolysis. The 1:1 binding kinetics and affinities were measured by surface plasmon resonance with Biacore. Clone AB1-C1 showed 4-fold improvement in binding dissociation constant (K_D_) when compared with parental AB1, whereas AB1-C16 had a smaller improvement in binding K_D_. The combination clone AB1-C1-16 behaved similarly to AB1-C1.

To evaluate the impact of these modest affinity improvements, we carried out a CCR6 Chinese hamster ovary (CHO)-K1 β-arrestin G protein–coupled receptor functional assay. This assay allows direct measurement of the activation of muCCR6 by muCCL20, as well as the inhibition of this pathway by an inhibitor. Characterization of these AB1 variants revealed a shift in the antibody IC_50_ by AB1-C1, AB1-C16, and AB1-C1-16. Although AB1-C16 maintained about a 2-fold decrease in IC_50_ (i.e., 2-fold increase in inhibition potency), AB1-C1 and AB1-C1-16 conferred about a 4-fold decrease in IC_50_. This demonstrated that the improved affinity conferred by these two amino acid changes led to the enhanced inhibition of the ligand-receptor interaction.

### Structural insight of the affinity-improving mutations

Molecular mechanics techniques allow thousands of mutations to be made in a protein system but suffer from a lack of conformational sampling. To understand why AB1-C1 and AB1-C16 mutations improve affinity, we employed FEP combined with the enhanced sampling method replica exchange with solute tempering (REST), FEP+ [35], to calculate free-energy changes in an enhanced conformational sampling framework.

**Fig 8:**
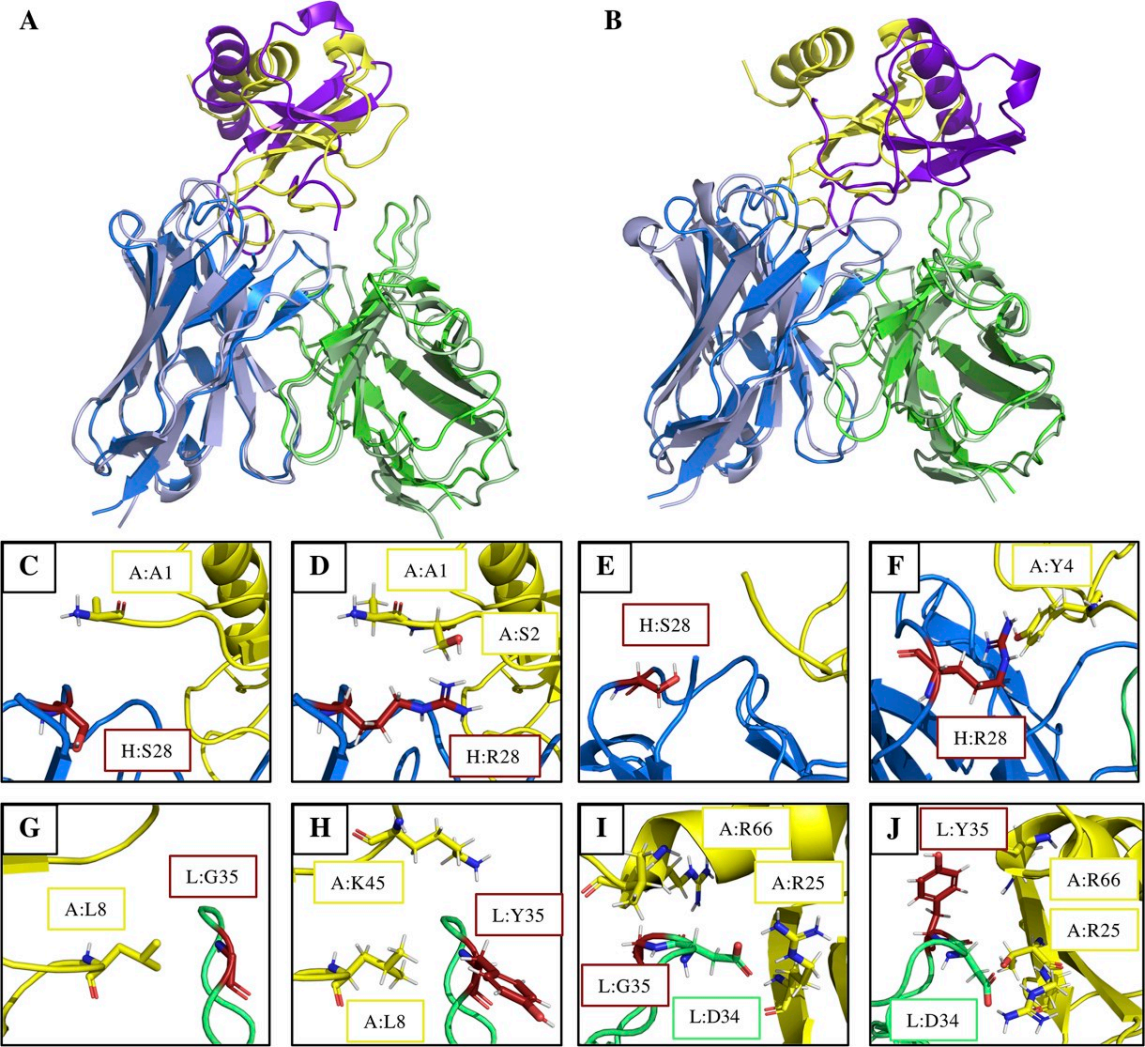
Comparison of minimised docked structure between AB1 and muCCL20 (V_H_, blue; V_L_, green; antigen, yellow) with (A) H:R28 and (B) L:Y35 FEP+ output structures (V_H_, light blue; V_L_, light green; muCCL20, purple). (C–J) Local structural environment of V_H_ (C–F) and V_L_ (G–J) mutation sites (red) in the docked conformation (C, G), MM conformations (D, H), FEP+ native (E, I) and FEP+ mutant conformations (E, H).

Both single mutations AB1-C1 and AB1-C16 showed improved affinities for the muCCL20 antigen, of –1.00 ± 0.26 kcal mol^-1^ (H:S28R) and –0.16 ± 0.06 kcal mol^-1^ (L:G35Y), respectively. This result echoed the greater affinity improvement of the AB1-C1 mutation over the AB1-C16 mutation that was observed in the binding ELISA. Inspection of both representative mutant structures indicated significant rearrangement (Fig 8A and 8B) in which “backbone” RMSDs of 2.6 Å and 2.4 Å were observed between the minimised input structure and the H:S28R and L:G35Y mutants, respectively.

Structural analysis of H:S28R showed that the introduction of arginine in the H:28 position facilitates the formation of a π-cation interaction between H:R28 and A:Y4 of the antigen (Fig 8F). As with the docked structure, (Fig 8C) enhanced sampling of the native structure (Fig 8E) indicates that there are no interactions present between L:S28 and muCCL20; hence the observed free energy change can be attributed to the introduction of a new intermolecular interaction. Interestingly, the mutation of L:G35 to tyrosine also introduces a new π-cation interaction between L:Y35 and R66 of the muCCL20. The presence of the salt bridge between the adjacent L:D34 residue and A:R25 (Fig 8H), which is not present in the docked conformation, can be explained through the representative conformation, i.e. the centroid of the most populated cluster, of the native state. The presence of the L:D34/A:R25 interaction in the native state, Fig 8I, indicates that the change in free energy cannot be assigned to the formation of a new salt bridge since it is present in both reference (native) and final states. Therefore, the modest binding free energy change can be accounted for by the π-cation interaction. The weaker change in ΔΔG_binding_ observed for L:G35Y compared to H:S28R is likely due to poorer interaction geometry, 5.7 Å/51.5°, compared to 4.9 Å/28.5° for H:R28/A:Y4.

Although both of these mutations were selected on the basis of MM ΔΔE_binding_ calculations (H:S28R, –9.07 AU; L:G35Y, –1.46 AU), the elucidation of these interactions is not possible by mapping the mutations onto the docked conformations (Fig 8C and 8G) or via molecular mechanics methods (Fig 8D and 8H) and are likely to be accessible only with enhanced sampling techniques.

## Discussion

*In silico* affinity maturation is a challenging yet promising area of protein engineering and antibody therapeutics. In this study, we chose a more unprecedented scenario, where the co-crystal structure of the antibody-antigen complex is not available with the wildtype antibody’s starting K_D_ in the low nanomolar range, to test out the potential of de-novo *in silico*-based affinity maturation. In this case study, careful consideration was given to every step in the process especially protein docking and the challenges it presents. We chose to confirm the docking poses using *in silico* and experimental alanine scanning mutagenesis to build confidence in the predictions. It is interesting to note that in the case of alanine scanning predictions, 14/23 mutations showed a reduction in binding, yet 9 mutations still retained binding when tested experimentally. This allowed us to hone in on the correct antibody-antigen interface to design affinity-improving mutations. Given the combination of potential errors from homology modelling, docking, and mutagenesis, it is remarkable that from a panel of only 19 variants, selected from three varying mutation protocols and energy functions, we were able to identify two affinity-improving mutations.

The adoption of three protocols, Discovery Studios, Schrödinger (MM-GBSA) and Rosetta (MM-LKSM), for affinity maturation proved useful in this study, since neither of the two hits that were identified were picked up by more than one method (Table 3). AB1-C1 (H:S28R) was discovered from the Schrödinger protocol and AB1-C16 (L:G35Y) came from Discovery Studio. Unlike the Assisted Design of Antibody and Protein Therapeutics (ADAPT) approach [11], which uses multiple protocols to find an inter-method consensus, thus strengthening the confidence in a particular mutation, we find that multiple methods provided greater breadth of mutational space and avoided a particular residue or site bias, albeit with a relatively limited data set. Despite the aforementioned challenges, the MM approach in this work was especially useful in the filtering of docked poses and the identification of key residues. Indeed, the novel utilisation of alanine scanning with experimental validation and the demonstrable necessity for redocking are significant findings of this work. *In silico* alanine scanning, however, may be expected to be more reliable than *in silico* affinity maturation, since engineering the loss of specific, high-energy interactions such as salt bridges is easier than introducing new gain-of-function mutations that may require significant structural rearrangement.

The approach with *in silico* affinity maturation outlined here has the potential advantage of generating significant savings in both time and resources for identifying potential hits. Based on our experience, a typical empirical affinity optimization campaign could take at least 6 months, whereas computational strategies coupled with focused experimental evaluations could generate significant time savings. In addition, without requiring the generation of antigen-antibody co-crystal structures, our approach saved around 3–18 months from a typical crystal structure-based *in silico* affinity maturation workflow. In addition, *in silico* design has the potential to streamline specificity and developability design parameters into the affinity-matured molecules.

Whilst our work highlights the synergy from combining computational and experimental approaches, we also acknowledge the need for significant development in this field. Conventional experimental random mutagenesis strategies (such as parsimonious mutagenesis or display library approaches), sometimes with the help of co-crystal structures, are highly effective for antibody affinity improvement and can often reach beyond 10- to 100-fold [3, 5, 36]. It has been much more challenging to reach that goal with a focused *in silico* design approaches based on crystal structures [9, 37–40], with even fewer successes in the absence of crystal structures of the antibody-antigen complex. A recently published case study demonstrated an improvement of the binding affinity of a single-domain intrabody to α-synuclein from micromolar affinity to 66 nM [41]. This work and ours have shown encouraging signs for advances in the *de novo* antibody affinity maturation space, but also show the challenges of pushing toward sub-nanomolar affinity. In addition, there is an opportunity to improve the positive hit rate from 2 out of 19 mutants, which is not much higher than what is observed in a random mutagenesis approach. We have shown that methods such as FEP+ can provide some progress; however, this method has not yet been optimised for biologics applications and can only predict impacts of single point mutations with some accuracy. It would also be prohibitively computationally costly to employ as a saturation mutagenesis approach, taking into account protein dynamics, side chain flexibility and physics-based free energy calculations. Utilising multiple MM-GBSA/MM-LKSM methods may also present an opportunity for advancement in this respect, acting as a pseudo-sampling approach in which the energy function of each method samples the conformation in a unique way and may reveal effects that are neglected by other protocols.

Here we present an interesting case study of *in silico* affinity optimisation in a scenario where co-crystal structure is not available. We demonstrate a novel strategy to achieve moderate affinity improvement using the integrated approach of *in silico* prediction and experimental guidance. Despite the limitations of the state of the technology that our work highlights, such as the need for more accurate energy prediction methods for large protein complexes, we hope to offer a path toward more advancement in the field of computational protein design and engineering.

## Materials and methods

### Antibody homology modelling

The AB1 Fv model was generated on February 15, 2017 with SAbPred [29], using AbodyBuilder [42] to search for a framework template structure, in this instance, 1MH5 for both V_H_ and V_L_ domains, from the curated antibody structure database. SabDab [43] and CDR loops were modelled with FREAD [44].

### Antibody-antigen docking

The nuclear magnetic resonance solution structure of muCCL20 was obtained from the protein data bank (PDB ID, 1HA6) [45]. The structural ensemble consisted of 20 low-energy structures, the first of which was representative of the average structure of the ensemble and was used for all subsequent *in silico* experiments.

The antibody Fv and muCCL20 structures were imported into the Discovery Studio 2016 software package [32], and both structures were prepared with the clean protein tool and subsequently typed with the CHARMM-Polar-H force field [46] prior to docking. Rigid-body docking was performed with ZDOCK [47], using an angular step size of 15° and a distance cut-off of 10 Å, and non-IMGT CDR residues of the antibody were blocked. Subsequent refinement was performed with RDock [47].

After alanine scanning mutagenesis and binding studies were performed, the antibody and antigen were redocked. The same protocol was employed, but with the additional parameters that all six AB1 residues that were identified as important for binding must be within 10 Å of muCCL20.

### *In silico* mutagenesis

*In silico* alanine scanning was initially performed on each of the five top candidate poses. Mutagenesis was performed in the Discovery Studio 2016 software package, and each mutant structure was minimised prior to ΔΔE calculations. Mutagenesis was also performed on the redocked conformations, again using the same Discovery Studio protocol employed for alanine scanning, in which 10 single-point mutations were performed on each of the 1925 output poses.

### *In silico* affinity maturation with three methods

#### Discovery studio protocol

The complex structure was applied with the CHARMM-Polar-H force field in the Discovery Studio 2016 GUI. The typed structure was then exported and used as the basis for mutagenesis. Using a Perl script specific to Discovery Studio, we mutated the hybrid CDR residues to all 20 genetically encoded amino acids and calculated both the ΔΔE_binding_ and ΔΔE_stability_ values, which resulted in 1400 mutations.

#### Schrödinger biologics suite protocol

The complex structure was prepared by using the Protein Preparation Wizard tool [48, 49] within the Schrödinger Biologics Suite 2016–3 [33]. The titratable residues were protonated in accordance with their environment by using PropKa [50, 51] and were typed with the OPLS3 [52] force field, and the structure was subsequently minimised.

Because optimisation of the wider environment of a mutation may result in the unwarranted acceptance of large side chains, two separate mutagenesis experiments were performed. One experiment used a 0.0 Å optimisation distance, that is, only the mutated reside, and one also minimised any residues within 5.0 Å. By using both optimisation criteria and taking a consensus of accepted mutations, it was possible to avoid selection of residues that sterically disrupted the local protein environment whilst identifying those that required only a small structural rearrangement to enter a conformation, thereby leading to improved binding affinity.

#### Rosetta protocol

Prior to mutagenesis, the complex structure was cleaned using the clean_pdb.py tool within Rosetta [34] and minimised using the Relax protocol to negate steric clashes. The 500 minimised structures were ranked by their total score, and the structure with lowest overall energy was used for mutagenesis.

Mutagenesis was performed with a Rosetta scripts protocol, which was used to optimise the mutation at each individual CDR position. In the mutagenesis.xml (S1 Text) the ΔΔE_stability_ filter is set to 0.0, and therefore only mutations that do not destabilise the complex or the interface are accepted. One hundred structures were generated for each site, and the most-stabilising mutations were identified by extracting the best score from the score.sc file and comparing the corresponding PDB with the native protein. In addition to the ΔΔE_binding_ calculations described in the “Results” section (see “*In silico* affinity maturation”), the Rosetta pmut (point mutation) protocol was employed to predict ΔΔE_stability_ values for all mutations.

#### Mutational comparison with WaterMap

WaterMap [53] analysis was performed on the parent complex structure, using Schrödinger Biologics Suite 2017–4. The structures of the 87 single mutations were taken from the Schrödinger 5.0 Å refinement protocol and were mapped onto the WaterMap results. Visual inspection was performed to determine whether each mutant could displace one or more WaterMap water molecules; if so, the ΔG values of each of the displaced molecules were combined.

#### Structural rationalisation of improved affinity mutations

FEP+, which is a combination of FEP [15] and the enhanced sampling method REST [54–56], was employed for post- rationalisation of the two affinity-improving mutations in addition to the double mutation. The combination of FEP and REST allows the system to explore more conformational states upon mutation and should provide a more reliable estimate of free-energy and structural changes.

FEP+ calculations were performed in Schrödinger Biologics Suite 2017-4 [57], using a simulation time of 20 ns to ensure free-energy convergence.

#### Mutant panel construction, expression, and purification

All chemicals were of analytical grade. Oligonucleotides were purchased from Eurofins MWG Operon (Louisville, KY). A plasmid-encoding monoclonal antibody, AB1, was generated with the In-Fusion HD cloning kit from Takara Bio (Mountain View, CA), encoding V_H_ and V_L_ sequences into an in-house immunoglobulin G1 mammalian expression vector. Point mutations were introduced by site-directed mutagenesis, using the QuikChange Multi Lightning mutagenesis kit (Agilent Technologies, Santa Clara, CA). All antibody positions are listed according to the IMGT numbering for the variable domains [31] and the European Union numbering convention for the C_H_2-C_H_3 domain [58].

The variants were transiently transfected into human embryonic kidney cell line HEK293FT, using 293Fectin transfection reagent (Life Technologies, Carlsbad, CA). Cells were grown in FreeStyle 293-F Expression Medium (Life Technologies). The expressed antibodies were purified from cell supernatant by affinity chromatography, using a HiTrap Protein A column (GE Healthcare Life Sciences, Marlborough, MA). Antibody was eluted with Pierce IgG Elution Buffer (Thermo Scientific, Waltham, MA) and neutralized with 1 M tris(hydroxymethyl)aminomethane (Tris), pH 8.0. Antibodies were dialyzed into phosphate-buffered saline (PBS), pH 7.2. Monomer content for all the antibodies was determined by analytical size exclusion chromatography to be greater than 95%.

#### Binding ELISA

Recombinant human and murine CCL20 were generated in-house by standard mammalian cell expression protocols [59]. For evaluation of species cross-reactivity, both mouse and human CCL20 were coated at 2 μg/mL onto Nunc Maxisorp 96-well plates (Thermo Fisher Scientific, Waltham, MA) and incubated overnight at 4°C. After five washes with PBS buffer with 0.1% Tween 20 (PBST), the plates were blocked with 3% bovine serum albumin (BSA) in PBST. The blocking buffer was decanted and antibody dilutions starting at 60 nM were then added to the wells. After five washes in PBST, secondary antibodies, horseradish peroxidase–conjugated goat anti-mouse Fc (Jackson ImmunoResearch, West Grove, PA) and anti-human Fc–horseradish peroxidase (Sigma-Aldrich, St. Louis, MO), were added at a 1:10,000 dilution and incubated for 1 h at room temperature. Binding was detected with the addition of SureBlue 3,3′,5,5′-tetramethylbenzidine (TMB) substrate (KPL, Gaithersburg, MD), and the reaction was stopped by adding TMB Stop Solution (KPL). Absorbance signals were read at 450 nm. For binding comparisons among AB1 variants, Nunc Maxisorp 96-well plates (Thermo Fisher) were coated with anti-mouse Fc antibody (Jackson Laboratories, Bar Harbor, ME) at 1 μg/mL in PBS (pH 7.2) and incubated overnight at 4°C. After three washes, the wells were blocked with 3% BSA in PBST. The blocking buffer was decanted, and antibody variants diluted to 5 μg/mL in PBS were added and incubated for 1 h at room temperature. After three washes, titrations of biotinylated muCCL20 from 0 to 600 nM were added to the plates and incubated for 1 h for binding.

#### Fab binding affinity surface plasmon resonance measurements

Purified antibodies were cleaved with immobilized papain for 5 h at 37°C, followed by incubation for 12 h at room temperature. The cleaved Fab fragments were purified by flow-through anion exchange chromatography, followed by acidification and cation exchange chromatography. The purified Fab molecules were analysed and confirmed by mass spectrometry. Kinetic rate constants (k_on_ and k_off_) for binding of the anti-muCCL20–Fab fragments from clones AB1-C1, AB1-C16, and AB1-C1-16 combined with purified muCCL20 proteins were measured by surface plasmon resonance, using a BIAcore T200 instrument (GE Healthcare Life Sciences). The muCCL20 protein was immobilized on a CM4 sensor chip, using a target surface density of ~30 resonance units. A reference flow cell surface was also prepared on this sensor chip by use of the identical immobilization protocol, but without muCCL20. Three-fold serial dilutions of all Fab molecules (0.02–50 nM) were made in instrument buffer (HBS-EP buffer: 0.01 M HEPES [N-2-hydroxyethylpiperazine-N'-2-ethanesulfonic acid], pH 7.4; 0.15 M NaCl; 3 mM ethylenediaminetetraacetic acid; and 0.005% P-20). For kinetic measurements, each concentration of Fab fragment was first injected over the immobilized muCCL20 and reference surfaces at a flow rate of 100 μL/min for 120 s. The resulting binding response curves yielded the association phase data. After the injection of Fab fragment, the flow was switched back to instrument buffer for 11 min to permit the collection of dissociation phase data, followed by a 1-min pulse of 10 mM glycine, pH 1.5, to regenerate the muCCL20 immobilized surface on the chip. Binding responses from duplicate injections of each concentration of Fab were recorded against muCCL20. In addition, several buffer injections were interspersed throughout the injection series. Selected buffer injections were used along with the reference cell responses to correct the raw data sets for injection artifacts and/or nonspecific binding interactions. Fully corrected binding data were then fit to a 1:1 binding model (Biacore T200 Evaluation Software version 2.0; GE Healthcare) that included a term to correct for mass transport-limited binding, should it be detected. These analyses determined the kinetic rate constants, k_on_ and k_off_, from which the apparent K_D_ was calculated as k_off_/k_on_.

#### In vitro muCCL20 activity

For antibody inhibition of the muCCL20 activation via muCCR6 receptor on the cell surface, the DiscoverX PathHunter CHO-K1 mCCR6 β-arrestin assay kit was used (Eurofins, Fremont, CA). Briefly, the transmembrane receptor CCR6 is expressed on CHO-K1 cells, and upon ligand activation, β-arrestin is intracellularly recruited to engage with the receptor, forcing complementation of the two β-galactosidase enzyme fragments. The resulting functional enzyme hydrolyses substrate to generate a chemiluminescent signal. Pretreatments were prepared in 96-well, U-bottomed plates with 4 nM of muCCL20 in CP-3 buffer and antibody variants at 1:3 serial dilutions. Media were decanted from cells in 96-well, white-walled plates. The plate was gently blotted dry, and 100 μL of pre-incubated treatments per well were transferred. DiscoverX detection reagent was prepared at 6 mL per plate. After 90 min of incubation at 37°C, 50 μL of detection reagent per well was added to β-arrestin assay plates, which were then incubated in the dark at room temperature for 60 min and read on an EnVision plate reader (Perkin Elmer, Waltham, MA) according to the manufacturer’s instructions.

## Supporting information

S1 Table *In silico* predicted change in binding affinity upon murine to human mutation of CCL20. (TIF)

S2 Table Change in binding energy of redocked poses. The ΔΔEbinding values (A.U.) of the 10 mutations for the 8 poses which qualitatively matched the loss, reduction or neutral impact on binding observed experimentally. Deleterious mutations are shown in red, the partial knock-out mutation L:K36A is shown in blue and neutral, control mutations are shown in green.
(TIF)

S1 Text Rosetta design.xml protocol. (DOCX)
