# Supplementary figures and images for: Experimentally guided computational antibody affinity maturation with de novo docking, modelling and rational design
Source: PLoS Comput Biol. 2019 May 1;15(5):e1006980. doi: 10.1371/journal.pcbi.1006980 (PMC6513101; doi:10.1371/journal.pcbi.1006980)

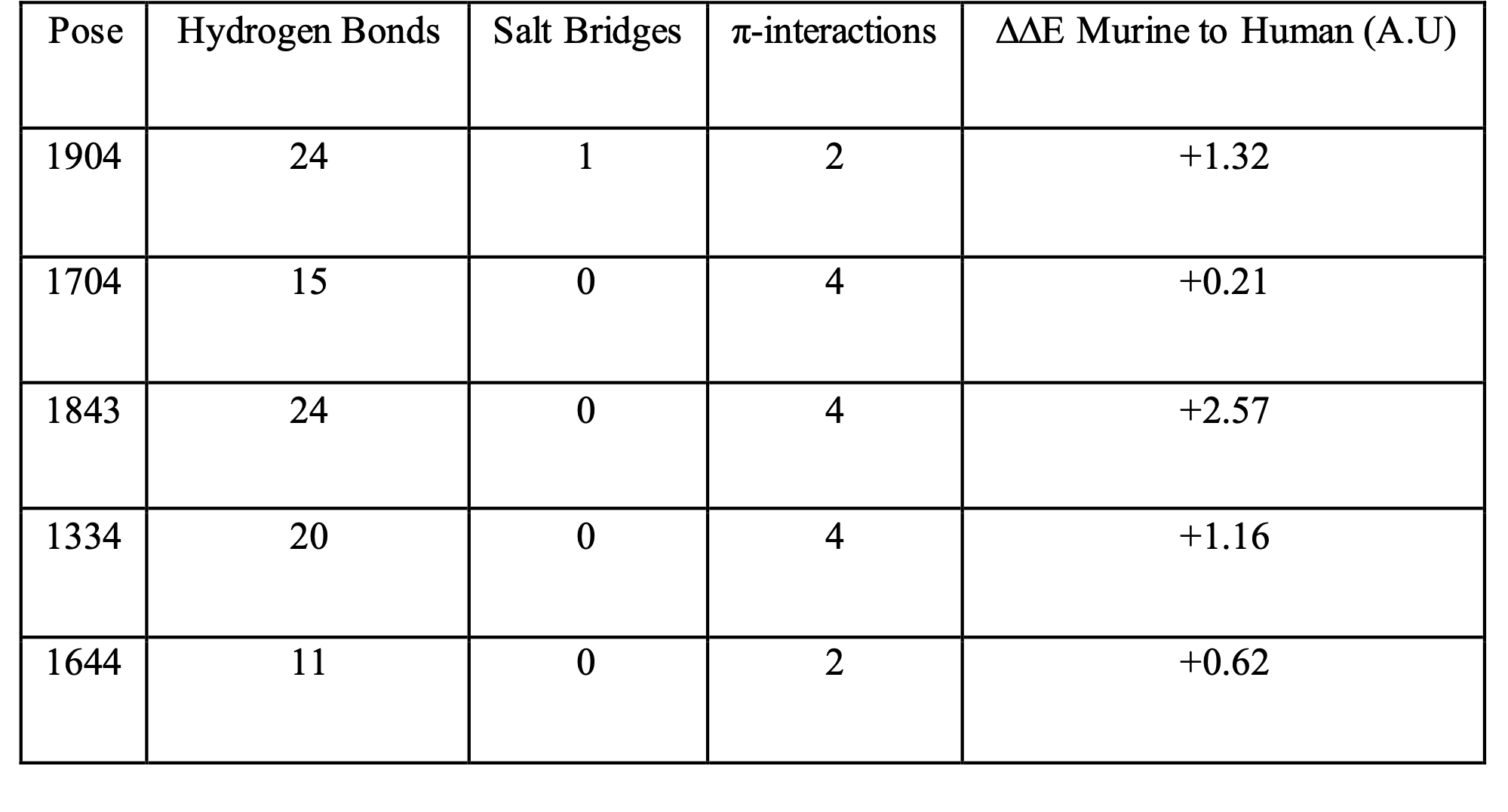

Supplement: S1 Table — (TIF) [file pcbi.1006980.s001.tif]

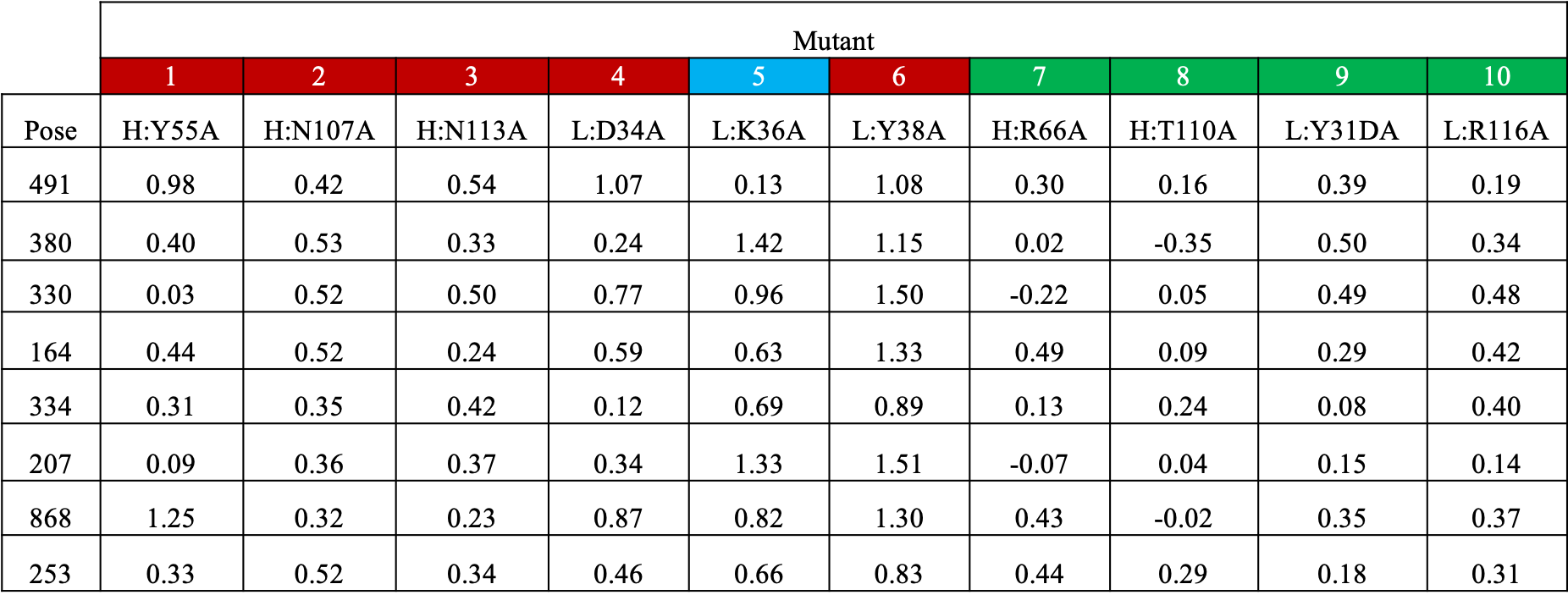

Supplement: S2 Table — The ΔΔEbinding values (A.U.) of the 10 mutations for the 8 poses which qualitatively matched the loss, reduction or neutral impact on binding observed experimentally. Deleterious mutations are shown in red, the partial knock-out mutation L:K36A is shown in blue and neutral, control mutations are shown in green. (TIF) [file pcbi.1006980.s002.tif]
