# Supplementary material for: Experimentally guided computational antibody affinity maturation with de novo docking, modelling and rational design
Source: PLoS Comput Biol. 2019 May 1;15(5):e1006980. doi: 10.1371/journal.pcbi.1006980 (PMC6513101; doi:10.1371/journal.pcbi.1006980)
Supplement: S1 Text — (DOCX) [file pcbi.1006980.s003.docx]

**S1 Text. Rosetta design.xml protocol**

<ROSETTASCRIPTS>

<SCOREFXNS>

</SCOREFXNS>

<FILTERS>

<Ddg name="ddg_filter" threshold="-5000.0" repeats="3" jump="2"/>

</FILTERS>

<TASKOPERATIONS>

<RestrictToRepacking name="repack_only" />

<ReadResfile name="resfile" filename="res.resfile"/>

</TASKOPERATIONS>

<MOVERS>

<PackRotamersMover name="pack" task_operations="repack_only"/>

<PackRotamersMover name="mut_and_pack" task_operations="resfile"/>

<FilterReportAsPoseExtraScoresMover name="dg_wt" report_as="ddg_wt" filter_name="ddg_filter" />

<FilterReportAsPoseExtraScoresMover name="dg_mut" report_as="ddg_mut" filter_name="ddg_filter" />

</MOVERS>

<PROTOCOLS>

<Add mover_name="pack"/>

<Add mover_name="dg_wt"/>

<Add mover_name="mut_and_pack"/>

<Add mover_name="dg_mut"/>

</PROTOCOLS>

</ROSETTASCRIPTS
